# Supplementary material for: Actin stress fiber organization promotes cell stiffening and proliferation of pre-invasive breast cancer cells
Source: Nat Commun. 2017 May 16;8:15237. doi: 10.1038/ncomms15237 (PMC5440822; doi:10.1038/ncomms15237)
Supplement: Supplementary Information — Supplementary Figures, Supplementary Tables and Supplementary References [file ncomms15237-s1.pdf]

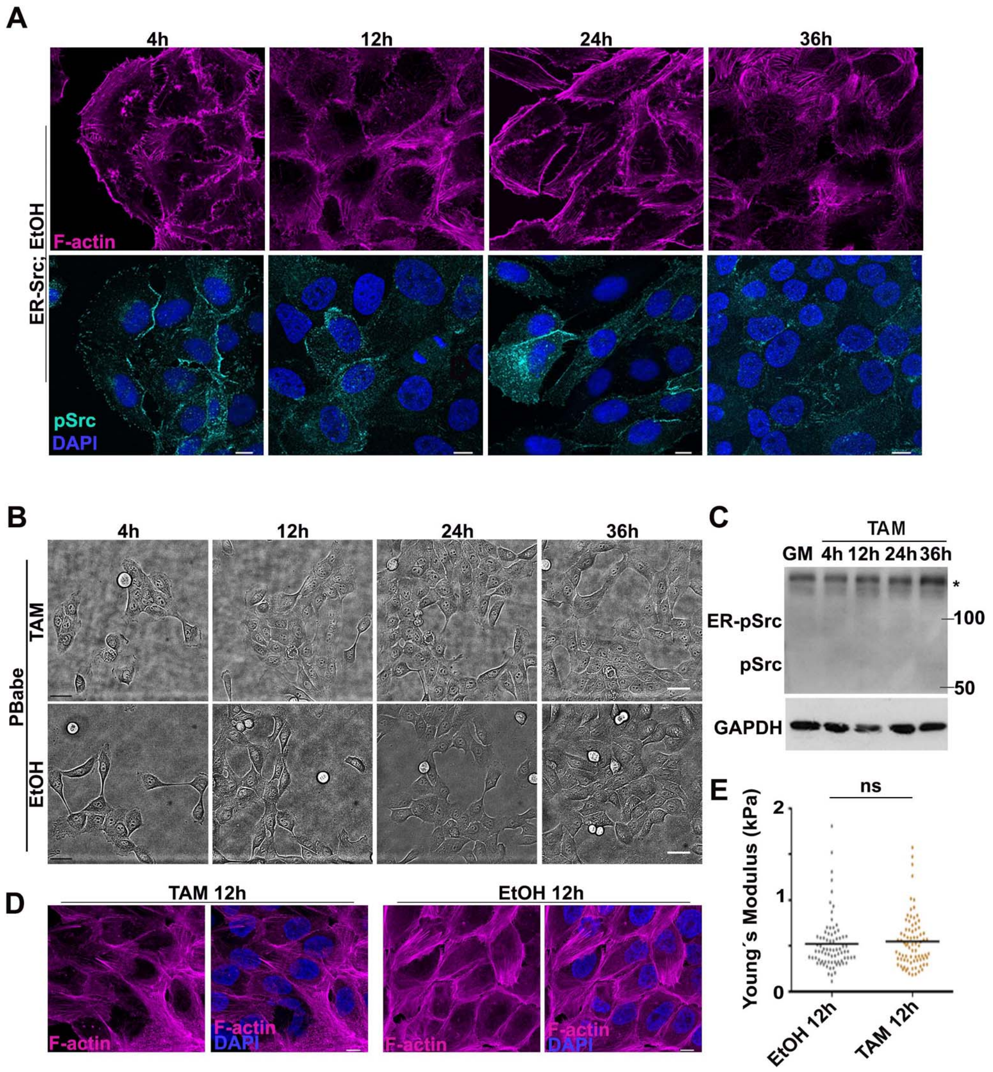

Supplementary Figure 1: Effects of TAM or EtOH on PBabe or ER-Src cells. (A) Standard confocal sections of ER-Src cells treated with EtOH for 4, 12, 24 and 36 hours, stained with Phalloidin (magenta), which marks F-actin, anti-pSrc (Cyan) and DAPI (blue). Scale bars represent 10 µm. **(B)** Images by phase contrast microscopy of PBabe cells, treated with EtOH or TAM for 4, 12, 24 and 36 hours. Scale bars represent 50 µm. **(C)** Western blot on protein extracts from untreated PBabe cells (GM) or PBabe cells treated with TAM for 4, 12, 24 or 36 hours, blotted with anti-pSrc or anti-GAPDH. The asterisk indicates non-specific bands revealed by the anti-pSrc antibody. **(D)** Standard confocal sections of PBabe cells 12 hours after EtOH or TAM treatments, stained with Phalloidin (magenta), which marks F-actin and DAPI (blue). Scale bars represent 10 µm**. (E)** Apparent Young´s modulus of PBabe cells 12 hours after EtOH or TAM treatments. The data points represented as dots with horizontal line indicating median values are from one biological replicate. ns indicates non-significant. Statistical significance was calculated using simple Mann Whitney tests. Related to Figures 1 and 2.

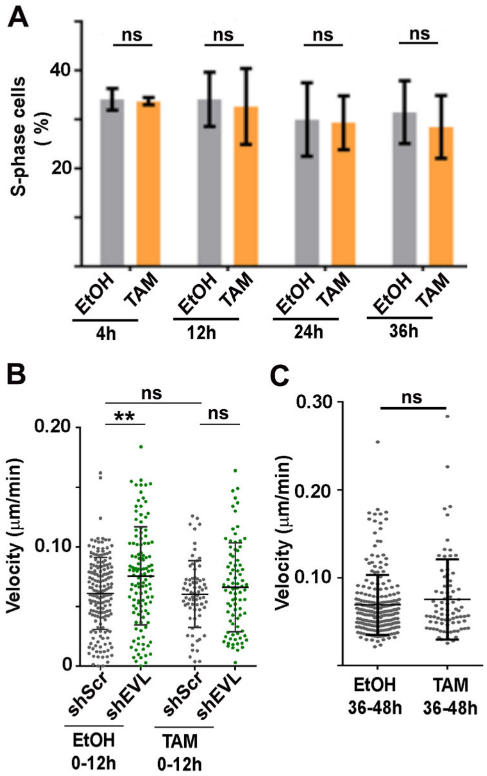

Supplementary Figure 2: Effects of Src on proliferation and migration. (A) Percentage of ER-Src cells in S-phase after treatment with EtOH (gray bars) or TAM (orange bars) for 4, 12, 24 or 36 hours in the presence of EGF. **(B)** Quantification of cell velocity during the first 12 hours of EtOH or TAM treatments in ER-Src cells expressing *shScr* (gray dots) or *shEVL* (green dots). **(C)** Quantification of ER-Src cell velocities between 36 and 48 hours of EtOH or TAM treatments. All quantifications were from three biological replicates. Error bars indicate SD. ns indicate non-significant. ** indicates P<0.001. Statistical significance was calculated using **(A)** two-way ANOVA or **(B)** one-way ANOVA or **(C)** impaired t-test. Related to Figure 1.

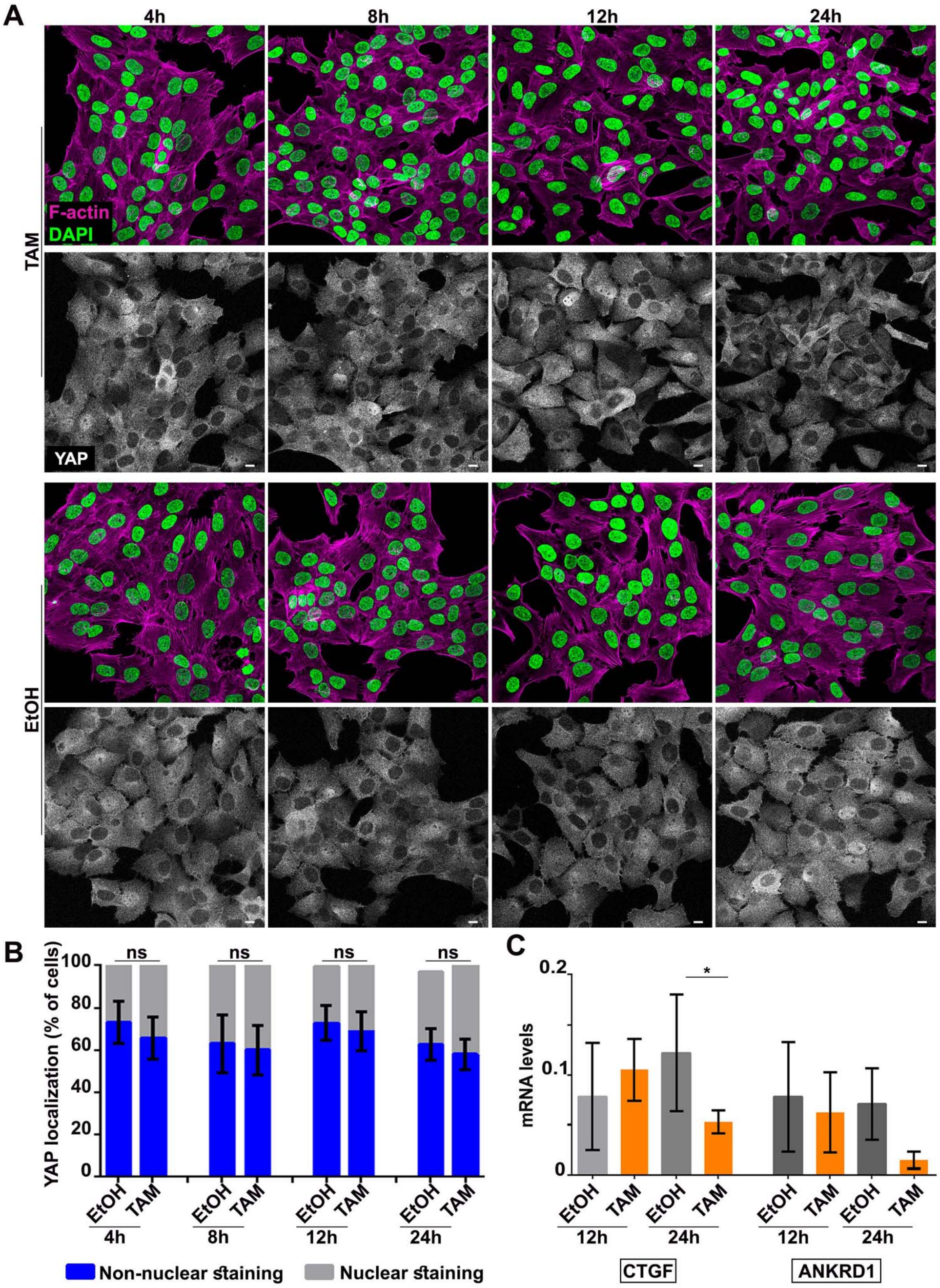

Supplementary Figure 3: YAP/TAZ activity in TAM-treated ER-Src cells. (A) Standard confocal sections of ER-Src cells treated with EtOH or TAM for 4, 8, 12, or 24 hours, stained with Phallodin (magenta) to mark F-actin, anti-p-YAP (Gray) and DAPI (Blue). Scale bars represent 10 µm. **(B)** Quantifications from two biological replicates of the percentage (%) of ER-Src cells in which YAP/TAZ is restricted to the cytoplasm (blue) or localizes to the nucleus (gray) after treatment with EtOH or TAM for 4, 8, 12 and 24 hours **(C)** Quantifications from three biological replicates of *CTGF* or *ANKRD1* mRNA levels measured by qRT-PCR on extracts from ER-Src cells treated with EtOH (gray bars) or TAM (orange bars) for 12 or 24 hours. Error bars indicate SD. ns indicates non-significant. * indicates $P<0.05$. Statistical significance was calculated using one-way ANOVA tests. Related to Figure 1.

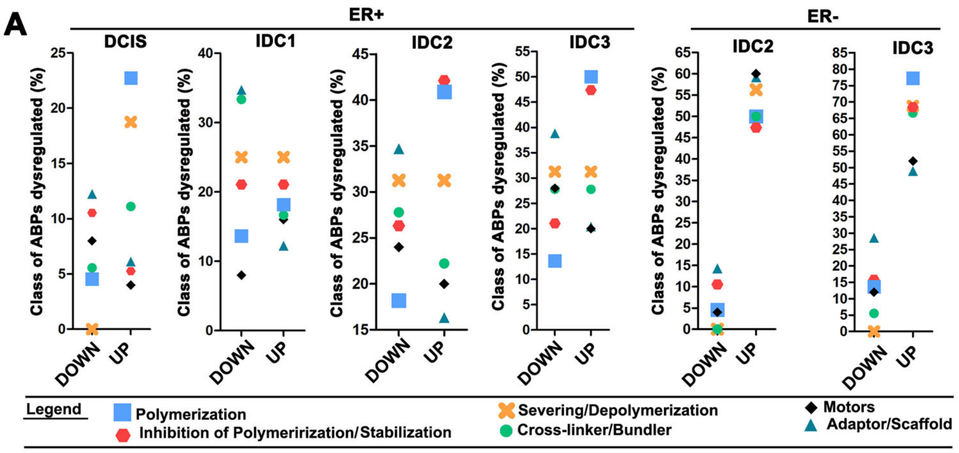

B

		Total	Down		Up	
	Polymerization	6	1	FHOD3	5	ARPC5L; ARPC3; ACTR3; ENAH; EVL
	Inhibition of polymerization/ Stabilization	3	2	TPM2; TMOD1	1	CAPZA1
	Filament severing/Depolymerization	3	0		3	MICAL2; CFL1; GMFB
	Cross-linkers/bundlers	3	1	TAGLN	2	MARCKSL1; PLS1
	Motors	2	2	MYO9A; MYH3	1	MYO6
	Adapter/ Scaffold	9	6	MYLK; PARVA; DST; SNTB2; CORO2B; SNTA1	3	LASP1; SHROOM2; EZR

Supplementary Figure 4: Classes of ABPs deregulated in breast lesions. (A) Percentage of functional classes of ABPs up- or down-regulated in ER^+ DCIS, ER^+ IDC1, ER^+ IDC2, ER^+ IDC3, ER^- IDC2 and ER^- IDC3 in comparison with normal breast tissues. ABPs were classified using the gene-ontology vocabulary as Polymerization (Blue, Square); Inhibition of polymerization/Stabilization (Red, Hexagon); Filament severing/Depolymerization (Yellow, Cross); Cross-linkers/ bundlers (Green, Dot); Motors (Black, Lozenge) and Adapter/Scaffold (Dark Blue, Triangle), according to their effect on actin (See also Supplementary Data 2). **(B)** Classification of the 27 ABPs misregulated in ER^+ DCIS, according to their functional role on actin and their expression levels (down- or upregulated) compared to normal breast tissues. Related to Figure 2.

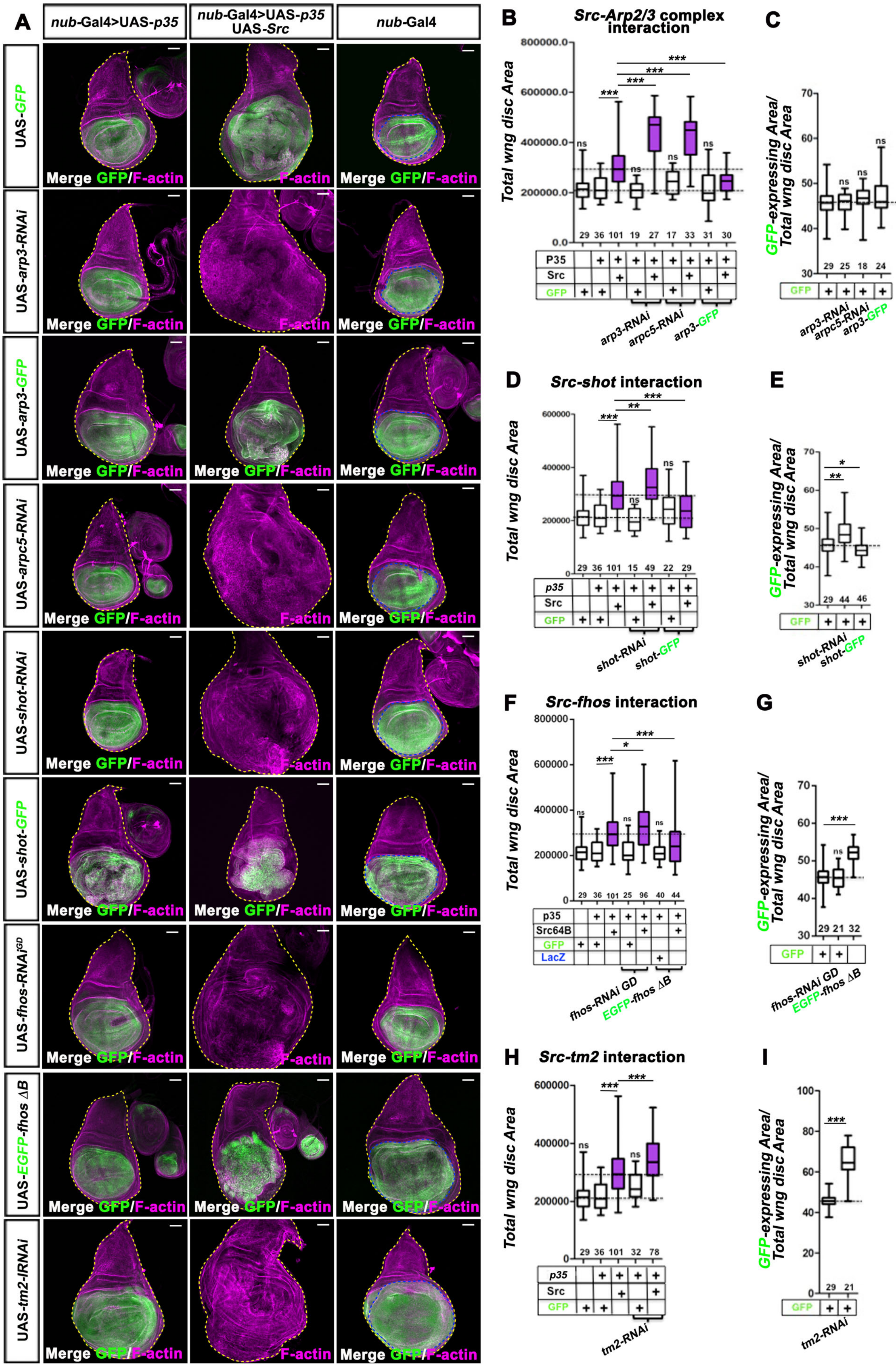

Supplementary Figure 5: Effect of *Drosophila* ABPs regulated by Src on tissue growth. (A) Standard confocal sections of third instar wing imaginal discs with dorsal side up, stained with Phalloidin to mark F-actin (magenta). *nub*-Gal4; UAS-*p35* or *nub*-Gal4; UAS-*p35*, *Src64B*^{UY133} or *nub*-Gal4 discs, carrying UAS-*mCD8-GFP* (green) or UAS-*arp3-RNAi*

or UAS-*arp3-GFP* (green) or UAS-*arpc5-RNAi* or UAS-*shot-RNAi* or UAS-*shot-GFP* (green) or UAS-*fhos-RNAi* or UAS-*EGFP-fhosΔB* (green) or UAS-*tm2-RNAi*. The yellow dashed lines outline the whole wing disc area. The blue lines outline the *nub>GFP*-expressing domain. Scale bars represent 30 µm. **(B-I)** Quantifications of total wing disc area (**B,D,F,H)** or ratio of the *nub>GFP* area over the total wing disc area **(C,E,G,I)** in discs in which Arp2/3 components **(B,C)**, or *shot* **(D,E)** or *fhos* **(F,G)** or *tm2* **(H,I)** expression are affected. Genotypes are indicated below each column. Magenta bars are nub>Src/p35-expressing wing discs in which the expression of Arp2/3 components or *shot* or *fhos* or *tm2* **is** affected. Numbers of samples from two biological replicates are indicated on the top of the X axis. ns indicates non-significant. Significance on the top of each column is for comparison with *nub>GFP, P35* **(B,D,F,H)** or *nub>GFP* **(C,E,G,I)**. Error bars indicate SD. *** indicates $P < 0.0001$. ** indicates $P < 0.005$. * indicates $P < 0.05$. Statistical significance was calculated using impaired t-test. Related to Figure 4**.**

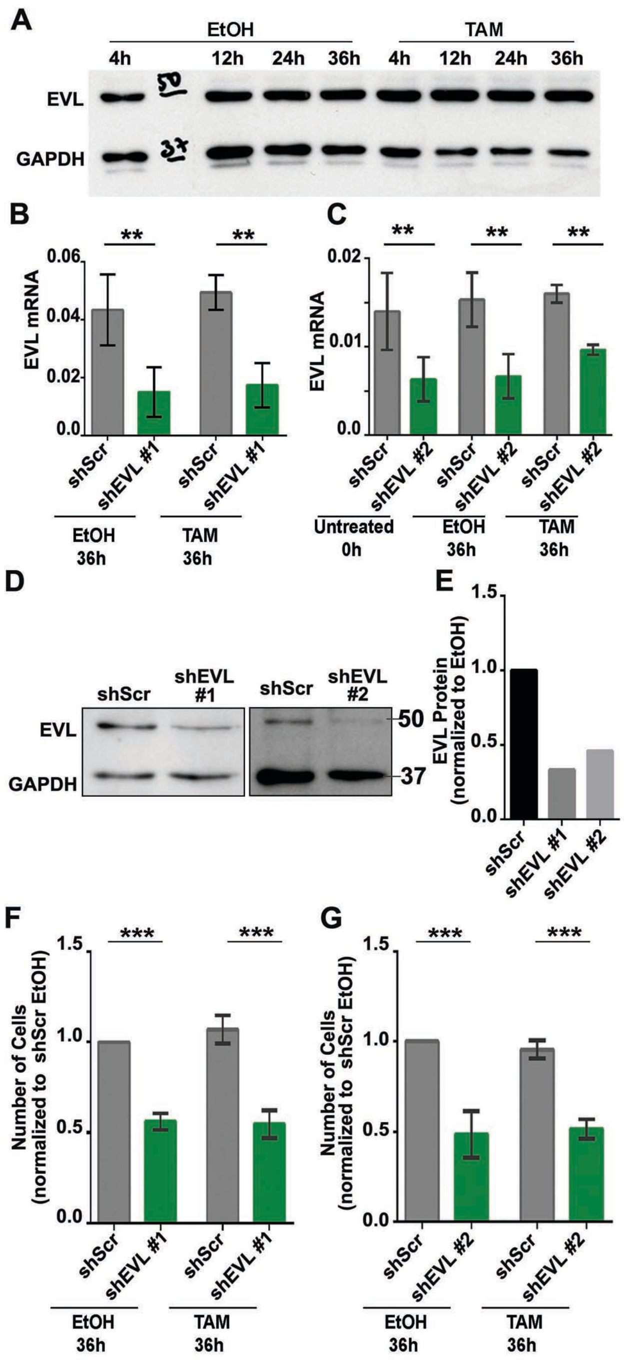

Supplementary Figure 6: EVL knocked down reduces cell growth. (A) Western blots on protein extracts from ER-Src cells treated with EtOH or TAM for 4, 12, 24 or 36 hours, blotted with anti-EVL and anti-GAPDH. **(B)** Quantifications from three biological replicates of *EVL* mRNA levels in extracts from ER-Src cells transfected with *shScr* (gray) or *shEVL#1* (green), and treated with EtOH or TAM for 36 hours. **(C)** Quantifications from three biological replicates of *EVL* mRNA levels on extracts from ER-Src cells expressing *shScr* (gray) or *shEVL#2* (green), untreated or treated with EtOH or TAM for 36 hours. **(D)** Western blot on protein extracts from ER-Src cells treated with *shScr* or *shEVL#1*, or *shEVL#2*, blotted with anti-EVL and anti-GAPDH. **(E)** Quantification from one biological replicate of total EVL levels in ER-Src cells treated with *shScr* (black bar) or *shEVL#1* (dark gray bar) or *shEVL#2* (light gray bar), normalized to GAPDH. **(F)** Quantifications from three biological replicates of the number of ER-Src cells expressing *shScr* (gray bars) or *shEVL#1* (green bars), treated with EtOH or TAM for 36 hours, normalized to EtOH-treated ER-Src cells expressing *shScr.* **(G)** Quantifications from three biological replicates of the number of ER-Src cells expressing *shScr* (gray bars) or *shEVL#2* (green bars), treated with EtOH or TAM for 36 hours, normalized to EtOH-treated ER-Src cells expressing *shScr*. Error bars indicate SD. ** indicates $P < 0.01$. *** indicates $P < 0.0001$. Statistical significance was calculated using one-way ANOVA tests. Related to Figure 5.

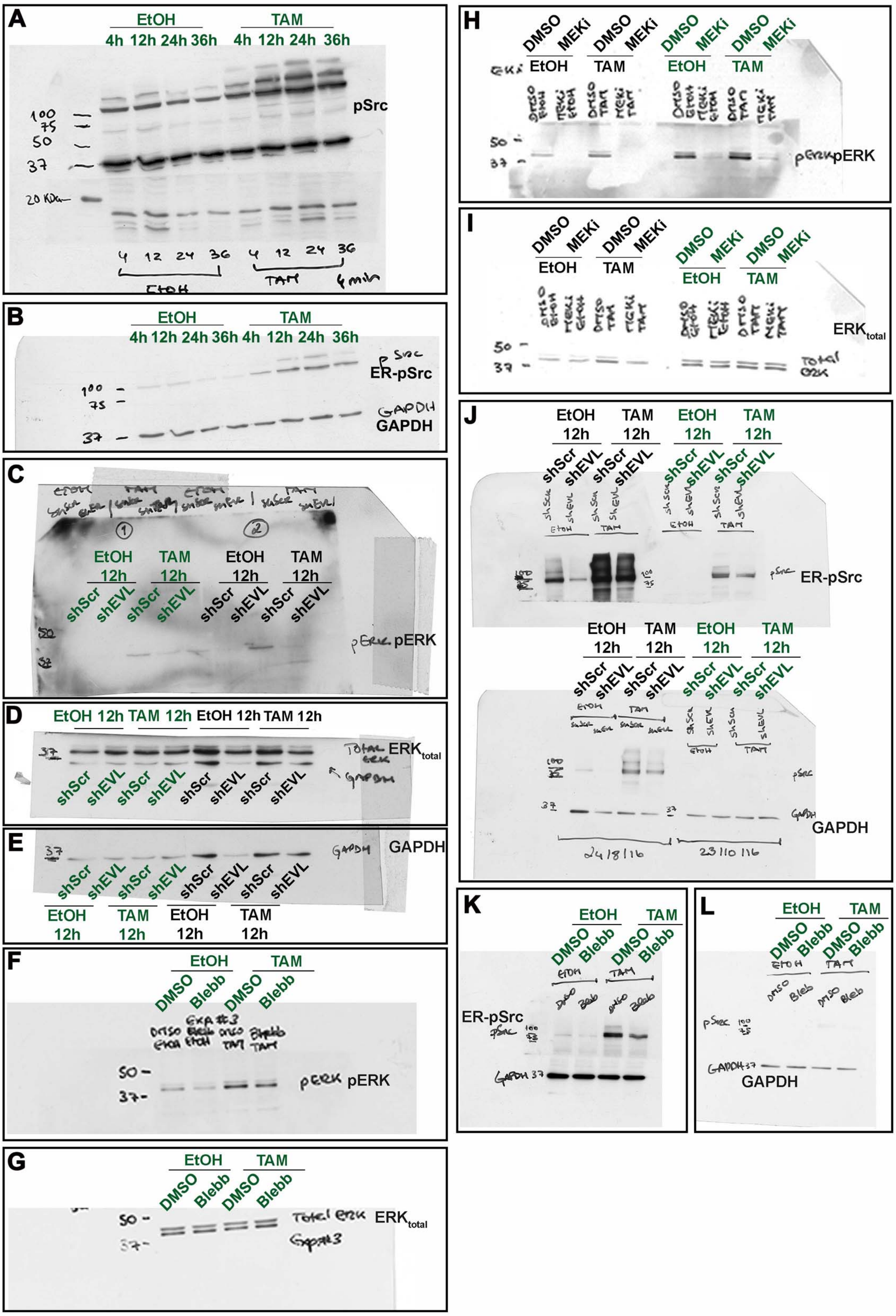

Supplementary Figure 7: Original images of the most relevant Western blots. **(A)** Western blot on protein extracts from ER-Src cells treated with EtOH or TAM for 4, 12, 24 or 36 hours, blotted with anti-pSrc, which reveals endogenous pSrc and ER-pSrc and anti-GAPDH. **(B)** lower exposure of the blot shown in A. See also Figure 1A. **(C-E)** Western blots on protein extracts from ER-Src cells expressing *shScr* or *shEVL#2* and treated with EtOH or TAM for 12 hours, blotted with **(C)** anti-pERK or **(D)** anti-ERK or **(E)** anti-GAPDH. See also Figure 5D. **(F-G)** Western blots on protein extracts from ER-Src cells treated with EtOH or TAM and DMSO or Blebbistatin for 12 hours, blotted with **(F)** anti-pERK or **(G)** anti-ERK. See also Figure 7B. **(H-I)** Western blots on protein extracts from ER-Src cells treated with DMSO or MEKi and EtOH or TAM for 12 hours, blotted with **(H)**

anti-pERK or **(I)** anti-ERK, which reveals total ERK. See also Figure 1G. **(J)** Western blots on protein extracts from ER-Src cells expressing *shScr* or *shEVL#2* and treated with EtOH or TAM for 12 hours, blotted with anti-pSrc or anti-pSrc and anti-GAPDH. See also Figure 5L. **(K-L)** Western blots on protein extracts from ER-Src cells treated with EtOH or TAM and DMSO and Blebbistatin for 12 hours, blotted with **(K)** anti-pSrc to reveal ER-pSrc or **(L)** anti-GAPDH. See also Figure 7F. Blots included in main figures are labeled in green.

Series	Platform	Samples											Reference
		N	ADH	DCIS	IDC								
					ER status		Grade division (IDC,ER+)			Grade division (IDC,ER-)			
					ER +	ER -	I	II	III	I	II	III	
**GSE15852**	HG-U133A	43	0	0	22	17	4	10	8	3	10	4	1
**GSE16873**	HG-U133A	12	12	12									2
**GSE7390**	HG-U133A	0	0	0	102	56	21	54	27		13	43	3
**GSE20194**	HG-U133A	0	0	0	115	93	7	64	44		13	80	4, 5
**GSE10810**	HG-U133A PLUS 2.0	27	0	0	11	11	2	7	2		2	9	6
**GSE21422**	HG-U133A PLUS 2.0	5	0	9									7
**GSE22544**	HG-U133A PLUS 2.0	4	0	0									8
**GSE23593**	HG-U133A PLUS 2.0	0	0	0	29	12	2	17	10			12	9
**GSE5460**	HG-U133A PLUS 2.0	0	0	0	44	50	15	11	18		5	45	10
**GSE5764**	HG-U133A PLUS 2.0	10	0	0									11
**GSE10780**	HG-U133A PLUS 2.0	143											12
**GSE2109**	HG-U133A PLUS 2.0	0	0	1	18	18	3	11	4	1	2	15	-
**GSE3744**	HG-U133A PLUS 2.0	7	0	0	15	24	0	0	15			24	13, 14
**GSE17907**	HG-U133A PLUS 2.0	4	0	0	13	20	1	4	8		3	17	15
**GSE19615**	HG-U133A PLUS 2.0	0	0	0	45	42	13	11	21		6	36	16
**GSE23177**	HG-U133A PLUS 2.0	0	0	0	116		0	0	116				17
**Total**		**255**	**12**	**22**	**530**	**343**	**68**	**189**	**273**				

Supplementary Table 1: Microarray platforms used in this study. The number of samples, their histological classification and associated references are indicated for each platform. N-normal tissue; ADH – atypical ductal hyperplasia; DCIS – ductal carcinoma *in Situ*; IDC – invasive ductal carcinoma of grade 1 (I) or grade 2 (II) or grade 3 (III). ER: Estrogen receptor. Related to Figure 3.

A

Human	*Drosophila*
EVL<br>ENAH<br>VASP	*enabled (ena)*
ACTR3<br>ACTR3B	*Actin-related protein 3 (Arp3)*
ARPC5L<br>ARPC5	*Actin-related protein 2/3 complex, subunit 5 (Arpc5)*
DST<br>MACF1	*short stop (shot)*
FHOD3<br>FHOD1	*formin homology 2 domain containing (fhos)*
TPM2<br>TPM3<br>TPM4<br>TPM1	*tropomyosin 2 (tm2)*

B

		Tissue growth (fold changes)			
		Src+; p35+		*wt*	
GFP		1		1	
ena	*RNAi*	0.75	*p<0.0001*	1.03	
	Over.	1.20	*p<0.0001*	1.06	*p<0.0009*
Arp3	*RNAi*	1.47	*p<0.0001*	1	
	Over.	0.82	*p<0.0004*	1.03	
Arpc5	*RNAi*	1.40	*p<0.0001*	1.03	
shot	*RNAi*	1.12	*p<0.005*	1.06	*p<0.001*
	Over.	0.80	*p<0.0002*	0.96	*p<0.03*
fhos	*RNAi*	1.08	*p<0.03*	0.99	
tm2	*RNAi*	1.18	*p<0.0001*	1.41	*p<0.0009*

Supplementary Table 2: Effect of ABPs deregulated by Src on tissue growth in the fly. (A) Human ABPs deregulated in TAM-treated ER-Src cells and in ADH/DCIS with their family members and their corresponding *Drosophila* counterpart. Orange and blue indicate genes up- and downregulated respectively. **(B)** Effect of knocking down (RNAi) or overexpressing (Over.) *ena, Arp3, Arpc5, shot, fhos* and *tm2* on growth of wing imaginal discs in which *nub*-Gal4 drives *Src64B*^{UY1332} and UAS-*p35* or UAS-*mCD8::GFP* (wt). Values represent fold changes relative to (first column) *nub>GFP, Src64BUY1332, p35* or (second column) *nub>GFP*. Blue and yellow indicate a significant suppression or enhancement relative to control, respectively. Related to Figure 4.

		EVL Classification - IDC		
		Count (%)		p-Value
		Neg	Pos	
Inflammation	0	10 (22.7)	5 (15.6)	0.037
	1	22 (50.0)	25 (78.1)	
	2	11 (25.0)	1 (3.1)	
	3	1 (2.3)	1 (3.1)	
	Total	44 (100)	32 (100)	
ER	Neg	29 (36.3)	6 (10.9)	0.037
	Pos	51 (63.8)	49 (89.1)	
	Total	80 (100)	55 (100)	
HER2	Neg	63 (78.8)	46 (83.6)	0.001
	Pos	17 (21.3)	9 (16.4)	
	Total	80 (100)	55 (100)	
CK5	Neg	75 (93.8)	54 (98.2)	0.479
	Pos	5 (6.3)	1 (1.8)	
	Total	80 (100)	55 (100)	
P-Cad	Neg	55 (68.8)	52 (94.5)	0.0002
	Pos	25 (31.3)	3 (5.5)	
	Total	80 (100)	55 (100)	
Subtype	Luminal A	50 (62.5)	46 (83.6)	0.008
	Luminal B	4 (5.0)	5 (9.1)	
	HER2 - OE	13 (16.3)	4 (7.3)	
	Basal	10 (12.5)	0 (0.0)	
	Ind.	3 (3.8)	0 (0.0)	
	Total	80 (100)	55 (100)	
Grade	Low	28 (35.0)	27 (49.1)	0.020
	Inter	22 (50.0)	25 (78.1)	
	High	11 (25.0)	9 (16.4)	
	Total	80 (100)	55 (100)	

Supplementary Table 3: Association of EVL expression in IDC. Association of EVL expression with clinicopathological features, including oestrogen receptor (ER) status, basal markers (CK5 and P-cad) expression, and breast carcinomas molecular subtypes in IDC. Related to Figure 8 and Table 1.

Supplementary References

1. Pau Ni, I.B. *et al.* Gene expression patterns distinguish breast carcinomas from normal breast tissues: the Malaysian context. *Pathol Res Pract* **206**, 223-228 (2010).
2. Emery, L.A. *et al.* Early dysregulation of cell adhesion and extracellular matrix pathways in breast cancer progression. *Am J Pathol* **175**, 1292-1302 (2009).
3. Desmedt, C. *et al.* Strong time dependence of the 76-gene prognostic signature for node-negative breast cancer patients in the TRANSBIG multicenter independent validation series. *Clin Cancer Res* **13**, 3207-3214 (2007).
4. Popovici, V. *et al.* Effect of training-sample size and classification difficulty on the accuracy of genomic predictors. *Breast Cancer Res* **12**, R5 (2010).
5. Shi, L. *et al.* The MicroArray Quality Control (MAQC)-II study of common practices for the development and validation of microarray-based predictive models. *Nat Biotechnol* **28**, 827-838 (2010).
6. Pedraza, V. *et al.* Gene expression signatures in breast cancer distinguish phenotype characteristics, histologic subtypes, and tumor invasiveness. *Cancer* **116**, 486-496 (2010).
7. Kretschmer, C. *et al.* Identification of early molecular markers for breast cancer. *Mol Cancer* **10**, 15 (2011).
8. Hawthorn, L., Luce, J., Stein, L. & Rothschild, J. Integration of transcript expression, copy number and LOH analysis of infiltrating ductal carcinoma of the breast. *BMC Cancer* **10**, 460 (2010).
9. Barry, W.T. *et al.* Intratumor heterogeneity and precision of microarray-based predictors of breast cancer biology and clinical outcome. *J Clin Oncol* **28**, 2198-2206 (2010).
10. Lu, X., Wang, Z.C., Iglehart, J.D., Zhang, X. & Richardson, A.L. Predicting features of breast cancer with gene expression patterns. *Breast Cancer Res Treat* **108**, 191-201 (2008).
11. Turashvili, G. *et al.* Novel markers for differentiation of lobular and ductal invasive breast carcinomas by laser microdissection and microarray analysis. *BMC Cancer* **7**, 55 (2007).
12. Chen, D.T. *et al.* Proliferative genes dominate malignancy-risk gene signature in histologically-normal breast tissue. *Breast Cancer Res Treat* **119**, 335-346 (2010).
13. Richardson, A.L. *et al.* X chromosomal abnormalities in basal-like human breast cancer. *Cancer Cell* **9**, 121-132 (2006).
14. Alimonti, A. *et al.* Subtle variations in Pten dose determine cancer susceptibility. *Nat Genet* **42**, 454-458 (2010).
15. Sircoulomb, F. *et al.* Genome profiling of ERBB2-amplified breast cancers. *BMC Cancer* **10**, 539 (2010).
16. Li, Y. *et al.* Amplification of LAPTM4B and YWHAZ contributes to chemotherapy resistance and recurrence of breast cancer. *Nat Med* **16**, 214-218 (2010).
17. Smeets, A. *et al.* Prediction of lymph node involvement in breast cancer from primary tumor tissue using gene expression profiling and miRNAs. *Breast Cancer Res Treat* **129**, 767-776 (2011).
